# Parliament and the Professions

**Published:** 1921-05-07

**Authors:** 


### Parliament and the Professions.

### Venereal Diseases Amongst Seamen.

In a question to the Minister of Health, Mr. Simm referred to the difficulty in obtaining treatment for venereal diseases experienced by British seamen in foreign ports. Sir Alfred Mond replied that the matter is under consideration by the Committee of the Office International d'Hygiene Publique, and will in all probability be dealt with by the International Health Organisation which is now established under the League of Nations.

### The Nurses' Co-operation.

Mr. Maclean asked the President of the Board of Trade whether he has given consideration to the complaints made against the Nurses' Co-operation, 22 Langham Street. Mr. Baldwin said that he had considered the matter, but his Department is awaiting further information which a former member of the staff of the Nurses' Co-operation has promised to supply.

